# Effects of long-term methylphenidate use on growth and blood pressure: results of the German Health Interview and Examination Survey for Children and Adolescents (KiGGS)

**DOI:** 10.1186/s12888-018-1884-7

**Published:** 2018-10-11

**Authors:** Suzanne McCarthy, Antje Neubert, Kenneth K. C. Man, Tobias Banaschewski, Jan Buitelaar, Sara Carucci, David Coghill, Marina Danckaerts, Bruno Falissard, Peter Garas, Alexander Häge, Chris Hollis, Sarah Inglis, Hanna Kovshoff, Elizabeth Liddle, Konstantin Mechler, Peter Nagy, Eric Rosenthal, Robert Schlack, Edmund Sonuga-Barke, Alessandro Zuddas, Ian C. K. Wong

**Affiliations:** 1 School of Pharmacy, University College Cork, Cork, Ireland; 2 Department of Paediatrics and Adolescents Medicine, University Hospital Erlangen, Erlangen, Germany; 3 Centre for Paediatric Pharmacy Research, Research Department of Practice and Policy, UCL School of Pharmacy, London, UK; 4 Department of Paediatrics and Adolescent Medicine, Li Ka Shing Faculty of Medicine, The University of Hong Kong, Hong Kong, Hong Kong; 5 Department of Medical Informatics, Erasmus University Medical Center, Rotterdam, Netherlands; 6 Department of Child & Adolescent Psychiatry and Psychotherapy, Medical Faculty Mannheim, Central Institute of Mental Health, University of Heidelberg, Mannheim, Germany; 7 Department of Cognitive Neuroscience, Donders Institute for Brain, Cognition and Behavior, Radboud University Medical Centre, & Karakter Child and Adolescent Psychiatry University Centre, Nijmegen, The Netherlands; 8 Child and Adolescent Neuropsychiatry Unit, Department of Biomedical Science, University of Cagliari & “A. Cao” Pediatric Hospital, Brotzu Hospital Trust, Cagliari, Italy; 9 Departments of Paediatrics and Psychiatry, Faculty of Medicine, Dentistry and Health Sciences, University of Melbourne, Melbourne, Australia; 10 Murdoch Children’s Research Institute, Melbourne, Australia; 11 Division of Neuroscience, School of Medicine, University of Dundee, Dundee, UK; 12 Department of Child and Adolescent Psychiatry, University Psychiatric Center, Leuven, KU Belgium; 13 Department of Neurosciences, University Psychiatric Center, Leuven, KU Belgium; 14 University Paris-Sud, Univ. Paris-Descartes, AP-HP, INSERM U1178, Paris, France; 15 Vadaskert Child and Adolescent Psychiatric Hospital, Budapest, Hungary; 16 Division of Psychiatry and Applied Psychology, Institute of Mental Health, School of Medicine, University of Nottingham, Nottingham, UK; 17 Tayside Clinical Trials Unit, University of Dundee, Dundee, UK; 18 Department of Psychology, University of Southampton, Southampton, UK; 19 Institute of Mental Health, Nottingham, UK; 20 Department of Paediatric Cardiology, Evelina Children’s Hospital, St Thomas’ Hospital, London, UK; 21 Unit of Mental Health Department of Epidemiology and Health Reporting, Robert Koch Institute, Berlin, Germany; 22 Department of Child and Adolescent Psychiatry, Institute of Psychiatry, King’s College London, London, UK; 23 Department of Experimental Clinical & Health Psychology, Ghent University, Ghent, Belgium; 24 Centre for Safe Medication Practice and Research, Department of Pharmacology and Pharmacy, Li Ka Shing Faculty of Medicine, The University of Hong Kong, Hong Kong, Hong Kong

**Keywords:** ADHD, Methylphenidate, Safety, Growth, BMI, Blood pressure

## Abstract

**Background:**

Concerns have been raised over the safety of methylphenidate (MPH), with regard to adverse effects on growth and blood pressure. Our study investigates whether, and to what extent, methylphenidate use in boys with ADHD is associated with having low body mass index (BMI), having low height, and increased systolic and diastolic blood pressure.

**Methods:**

Data used for this study stem from the German KiGGS dataset. Three different groups of boys aged 6–15 years were included in the analysis: ADHD patients who used MPH for less than 12 months; ADHD patients who used MPH for 12 months or more; and ADHD patients without current MPH treatment. Each of these three groups was compared to a non-ADHD control group regarding low weight (BMI ≤ 3rd percentile), low height (≤3rd percentile) and raised systolic and diastolic blood pressure. For growth outcomes, boys were categorized according to age (< 11 years/≥11 years, to account for pubertal maturation). Multivariable logistic regression was conducted to test for associations.

**Results:**

4244 boys were included in the study; MPH < 12 months: *n* = 65 (*n* = 36 < 11 years), MPH ≥ 12 months: *n* = 53 (*n* = 22 < 11 years), ADHD controls: *n* = 320 (*n* = 132 < 11 years), non-ADHD controls: *n* = 3806 (*n* = 2003 < 11 years). Pre-pubertal boys with MPH use less than 12 months and pubertal/postpubertal boys with MPH use of 12 months or greater were significantly more likely to have a BMI ≤ 3rd percentile compared to non-ADHD controls. Boys from the ADHD control group were significantly less likely to have a raised systolic blood pressure compared to non-ADHD controls. Beyond that, no significant between group differences were observed for any other growth and BP parameter.

**Conclusion:**

The analyses of the KiGGS dataset showed that MPH use in boys with ADHD is associated with low BMI. However, this effect was only observed in certain groups. Furthermore, our analysis was unable to confirm that MPH use is also associated with low height (≤3rd percentile) and changes in blood pressure.

## Background

Attention deficit hyperactivity disorder (ADHD) is characterized by pervasive and impairing hyperactivity, inattention and impulsiveness [1]. The worldwide prevalence of ADHD among school-aged children and adolescents is around 5% [2, 3]. Methylphenidate (MPH), the most commonly prescribed psychopharmacological treatment of ADHD in Europe, is effective at improving ADHD symptoms [4]. However, there have been reports in the literature concerning the effect of MPH on growth [5] and adverse cardiovascular outcomes [6].

### Height and weight

Effects on growth are usually reported as minor at the group level, but there is wide variability with some children unaffected [7–9] and others reporting moderate growth suppression [10–12]. Longitudinal studies suggest an overall height stunting of approximately 1 cm/year during the first three years of treatment which can be clinically relevant. These effects appear to attenuate over time and terminal adult height is not necessarily reduced [5, 13]. A Cochrane review published in 2015 examined the literature on the beneficial and harmful effects of methylphenidate on children and adolescents with ADHD [14]. With respect to the effect of methylphenidate on weight, five trials including 805 participants reported that children taking methylphenidate weighed significantly less than controls and six trials (859 participants) reported a decrease in weight among children taking methylphenidate. Children were also more likely to have a lower body mass index (BMI) based on the findings from one study with 215 participants [14]. However, disentangling these effects from growth deficits that may be associated with ADHD itself is challenging [5, 10, 15–17].

### Cardiovascular outcomes

A number of studies have investigated cardiovascular events (e.g. stroke, myocardial infarction, ventricular arrhythmia, sudden death) and cardiovascular effects (e.g. changes in BP and heart rate) associated with MPH.

Dalsgaard et al. [18] conducted a prospective longitudinal cohort study in Denmark (*n* = 714,258) to determine the risk of cardiovascular events among stimulant users. They reported a stimulant-related increase in risk for cardiovascular events (adjusted Hazard Ratio = 1.83 (1.10–3.04)) that persisted after restricting the analysis to only children with ADHD (*n* = 8300) (adjusted Hazard Ratio = 2.20 (2.15–2.24)).

A pooled analysis of three large data sets (19–21) including more than 1.8 million patients (ages 2–24 years [19], 25–64 years [20], 3–17 years [21]) reported no association between methylphenidate, amphetamine (AMP) and atomoxetine and sudden death or stroke (adjusted Odds Ratio = 0.93 (0.731.17)) [22]. However, in the study by Cooper et al. [19], it was reported that while there was no evidence of an association of serious cardiovascular events with ADHD drug use identified (adjusted Hazard Ratio = 0.70 (0.31–1.85)), “the upper limit of the 95% confidence interval indicated that a doubling of the risk could not be ruled out. However, the absolute magnitude of such an increased risk would be low”. A population-based, retrospective cohort study, conducted by Winterstein et al. [23], reported no significant association between central nervous stimulants and cardiovascular events (stroke, acute myocardial infarction and sudden cardiac death as a primary composite endpoint and the previous events plus ventricular arrhythmia as a secondary composite endpoint (adjusted Odds Ratio = 0.62 (0.27–1.44) and 0.74 (0.38–1.46) for the primary and secondary endpoints respectively [23]).

In terms of cardiovascular effects, stimulant medication can cause small increases in heart rate and BP. On average, heart rate increases of 1–2 beats per minute are reported at the group level, although larger increases can occur in some individuals. Average increases in systolic and diastolic BP range between 1 and 4 mmHg and 1 and 2 mmHg respectively [24]. Few studies report categorical hypertension data (defined as BP beyond the 95th centile) although anecdotally and from case reports, it is reported that MPH can cause a rise in BP above the 95th centile in some individuals [25].

The Committee for Medicinal Products for Human Use at the European Medicines Agency concluded in January 2009 that the overall benefits of MPH outweigh the risks; at the same time they highlighted the need for more data on the long-term effects of MPH on children and young adults as many of the trials conducted to date had focused only on short-term effects [26].

The aim of this study was to examine associations between MPH use in ADHD, body mass index (BMI) and height and BP in boys. Data from the German Health and Examination Survey for Children and Adolescents (KiGGS), a population-based German representative sample, were analyzed to evaluate the hypothesis that boys with ADHD and current MPH use show higher percentages of having low BMI, having low height, and of increased systolic blood pressure (SBP) and diastolic blood pressure (DBP) compared to controls.

## Methods

### Data source

Data for the present study stem from the KiGGS database. KiGGS was conducted by the Robert Koch Institute, Berlin, Germany, between May 2003 and May 2006. A two-stage sampling strategy was utilised: in the first stage, a representative sample of 167 German municipalities was identified. In the second stage, random samples of children and adolescents aged between 0 and 17, and stratified by sex and age, were then selected from these municipalities through local population registries. The resulting sample included 17,461 children and adolescents (8985 boys, 8656 girls). Detailed information on the KiGGS dataset and the sampling strategy have been reported elsewhere [27, 28].

### Sample selection

Individuals were classified as having ADHD if a parent reported that a diagnosis of ADHD had been made by a physician or psychologist [29]. Current ADHD-medication use (defined as use of medication within the seven days prior to interview) was documented using a standardized computer-assisted personal drug use interview which was conducted with the participants and their parents by a study physician [29]. The study design ensured that the study subjects were not interviewed during school holidays to exclude discontinuity of ADHD medication due to school breaks [30]. Duration of current medication use was categorized as either: less than 12 months or 12 months and longer. Patients with a record of current AMP use were excluded from the analyses in line with this study’s aim to focus on methylphenidate exclusively. Information on past use of medication was not recorded in the database. Patients who did not have a value recorded for a particular outcome variable (BMI, height, systolic blood pressure (SBP) and diastolic blood pressure (DBP)) were excluded from that specific variable analysis.

Four groups of individuals were identified for inclusion in the study: two groups who were taking MPH (< 12 months and ≥ 12 months), an ADHD group not currently taking MPH and a non-ADHD control group. Matching of controls to the medicated cohort was not undertaken. Preliminary analyses revealed that there were low numbers of females and older adolescents amongst the ADHD groups generally and the MPH groups specifically and so the study was restricted to males aged between 6 and 15 years inclusive.

### Outcome variables/ assessments

BMI, height: Body weight was measured in underwear to the nearest 0.1 kg with a calibrated scale (SECA, Birmingham, UK). Body height was measured by trained staff according to a standardized protocol to the nearest 0.1 cm using portable devices (standing height with Harpenden stadiometer for ages 2–17; Holtain Ltd., Crymych, UK). Measurement procedures were subject to internal and external quality control measures [31]. BMI was calculated as the ratio of weight (kg) to height (m) squared [31].

BP: Two readings of SBP and DBP were obtained using an automated oscillometric device (Datascope Accutorr Plus) at 2-min intervals after a non-strenuous part of the examination and an additional 5-min rest. The measurements were taken using the right arm, in the sitting position with the elbow at the level of the right atrium, using 1 of 4 cuff sizes which had to cover at least two-thirds of the upper arm length (from the axilla to the ante-cubital fossa). The mean of the two measurements was used for analysis [32]. The KiGGS dataset included a variable *cardiac disease* (code ca07); this variable, based on parents’ reports, was recorded in the dataset as ‘yes’, ‘no’ or ‘don’t know’.

### Outcome references

#### BMI and height

German reference data for BMI and height were obtained [31, 33]. The main focus of the data analyses was the percentage of boys with low BMI, defined as BMI <3rd percentile and the percentage of boys with a height ≤ 3rd percentile [34]. Analyses were conducted on the cohort as a whole (from 6 to 15 years) as well as according to two age categories, defined as 6–10 years and 11–15 years. By splitting the sample according to age, we use a simplified method that allows us to separate prepubertal children from peri and pubertal boys in the analyses, thus amplifying the power to detect an impact of MPH on growth.

#### BP

For the analysis of SBP and DBP, guidelines from the European Society of Hypertension [35] were used since these guidelines did not exclude overweight children unlike German reference data. (32).

Definition and classification of hypertension was as follows [35]:

**Table:** 

Class	SBP / DBP percentile
Normal	<90th
High-normal	≥90th to < 95th ≥120/80 even if below 90th percentile in adolescents
Stage 1 hypertension	95th percentile to the 99th percentile plus 5 mmHg
Stage 2 hypertension	>99th percentile plus 5 mmHg

Both high-normal BP (≥90th to <95th percentile) and hypertension (Stage 1 (95th percentile to the 99th percentile plus 5 mmHg) or Stage 2 hypertension (>99th percentile plus 5 mmHg)) were collapsed to form “Raised SBP” and “Raised DBP” categories for the purposes of the regression analyses.

#### Statistical methods

For each outcome variable, mean and standard deviations (sd) was calculated. Odds Ratios (OR) and 95% Confidence Intervals (95% CI) were derived from logistic regression analyses. Each of the three ADHD groups was compared to a non-ADHD control group regarding weight (BMI ≤ 3rd percentile), height (≤3rd percentile) and raised SBP and DBP.

For the outcomes BMI and height, boys were categorized according to age (< 11 years/≥11 years, to account for pubertal maturation). The relationship between blood pressure and MPH use was examined using logistic regression adjusting for age, BMI and cardiac disease. Sensitivity analysis was conducted removing patients who had a cardiac disease code of yes or don’t know.

A *p*-value of < 0.05 was considered significant for all analyses.

## Results

i) Sample characteristics:

A total of 4244 boys were included in the study: n = 65 in MPH < 12 months, *n* = 53 in MPH ≥ 12 months, *n* = 320 ADHD controls, and *n* = 3806 non-ADHD controls; 2193 (51.7%) boys of the total sample were 6–10 years old, 2051 boys (48.3%) were aged 11–15 years. For details see Table 1.

**Table 1:** Number (%) of boys in each study cohort

	MPH < 12 months	MPH ≥ 12 months	ADHD control	Non-ADHD control	Total (n)
Boys 6–10 years (n / %)	36 (0.85)	22 (0.52)	132 (3.11)	2003 (47.20)	2193
Boys 11–15 years (n / %)	29 (0.68)	31 (0.73)	188 (4.43)	1803 (42.48)	2051
Total (n / %)	65 (1.53)	53 (1.25)	320 (7.54)	3806 (89.68)	4244

### Body mass index

BMI data were available for 4229 boys (99.65%). Data of the group of boys aged 6–10 years (*n* = 2185) and those aged 11–15 years (*n* = 2044) were analyzed separately. The focus of the analyses was the percentage of boys who had low BMI, defined as BMI ≤ 3rd percentile. In the group of boys aged 6–10 years, those with MPH use of less than 12 months were significantly more likely to have a BMI ≤ 3rd percentile compared to the non-ADHD control group (OR 4.52 (95% CI, 1.54–13.28), *p* = 0.006). Whereas in the subsample of boys aged 11–15 years, those with MPH use ≥12 months were significantly more likely to have a BMI ≤ 3rd percentile compared to the non-ADHD control group (OR 3.59 (1.06–12.22), *p* = 0.040). Moreover, when compared to the non-ADHD control group no significant differences with regard to low weight were observed for the other groups (see Table 2).

**Table 2:** BMI data for 4229 boys

BMI ≤ 3rd percentile, n (%)	MPH < 12 months (*n* = 65)	MPH ≥ 12 months (*n* = 53)	ADHD control (*n* = 317)	Non-ADHD control (*n* = 3794)
Boys 6–15 years (*n* = 4229)				
Yes	5 (7.69)	4 (7.55)	11 (3.47)	108 (2.85)
No	60 (92.31)	49 (92.45)	306 (96.53)	3686 (97.15)
OR (95% CI), p-value	2.84 (1.12–7.22) 0.028	2.79 (0.99–7.89) 0.053	1.23 (0.65–2.31) 0.523	Reference
Boys 6–10 years (*n* = 2185)				
Yes	4 (11.11)	1 (4.55)	3 (2.29)	56 (2.81)
No	32 (88.89)	21 (95.45)	128 (97.71)	1940 (97.19)
OR (95% CI), p-value	4.52 (1.54–13.28), 0.006	1.83 (0.24–14.04), 0.561	0.84 (0.26–2.71), 0.766	Reference
Boys 11–15 years (*n* = 2044)				
Yes	1 (3.45)	3 (9.68)	8 (4.30)	52 (2.89)
No	28 (96.55)	28 (90.32)	178 (95.70)	1746 (97.11)
OR (95% CI), p-value	1.20 (0.16–8.98), 0.860	3.59 (1.06–12.22) 0.040	1.51 (0.71–3.23), 0.289	Reference

### Height

Height data were available for 4242 boys (99.95%). In accordance with the analysis of the BMI data we distinguished between boys aged 6–10 years and 11–15 years. In both age-groups, no significant differences between the MPH and ADHD control groups and the non-ADHD control group were observed with regard to the percentage of boys with a height ≤ 3rd percentile (see Table 3).

**Table 3:** Height data for 4242 boys

Height ≤ 3rd percentile, n (%)	MPH < 12 months (*n* = 65)	MPH ≥ 12 months (*n* = 53)	ADHD control (*n* = 319)	Non-ADHD control (*n* = 3805)
Boys 6–15 years (*n* = 4242)				
Yes	2 (3.08)	3 (5.66)	11 (3.45)	104 (2.73)
No	63 (96.92)	50 (94.34)	308 (96.55)	3701 (97.27)
OR (95% CI), *p*-value	1.13 (0.27–4.68) 0.866	2.10 (0.64–6.85) 0.219	1.25 (0.67–2.36) 0.483	Reference
Boys 6–10 years (*n* = 2191)				
Yes	2 (5.56)	2 (9.09)	3 (2.29)	53 (2.65)
No	34 (94.44)	20 (90.91)	128 (97.71)	1949 (97.35)
OR (95% CI), *p*-value	2.22 (0.52–9.53), 0.282	3.94 (0.88–17.62), 0.073	0.88 (0.27–2.86), 0.830	Reference
Boys 11–15 years (*n* = 2051)				
Yes	0 (0.00)	1 (3.23)	8 (4.26)	51 (2.83)
No	29 (100.00)	30 (96.77)	180 (95.74)	1752 (97.17)
OR (95% CI), *p*-value	-	1.19 (0.16–8.95), 0.863	1.55 (0.72–3.32), 0.260	Reference

### Blood pressure

Blood pressure data were available for 4238 boys (99.86%). Mean SBP and mean DBP were similar across the different groups. Stage 2 hypertension based on SBP and/or DBP was generally very rare and was not observed in boys currently taking MPH. However, stage 1 hypertension based on SBP and/or DBP could be observed most frequently in those taking MPH ≥ 12 months (for details see Table 4). The percentage of boys in the ADHD control group with raised SBP was statistically significantly lower than in the non-ADHD control group (OR 0.65 (95% CI 0.46–0.92), *p* = 0.016). Moreover, no statistically significant differences between the MPH groups and ADHD control groups and the non-ADHD control group with regard to raised SBP nor to raised DBP could be found (See Table 5). These findings persisted after removal of such patients with a cardiac disease code of *yes* or *don’t know*.

**Table 4:** Blood Pressure data for 4238 boys

	MPH < 12 months (*n* = 65)	MPH ≥ 12 months (*n* = 53)	ADHD control (*n* = 318)	Non-ADHD control (*n* = 3802)
i) Mean SBP (SD) / mmHg	107.71 (9.43)	107.23 (9.48)	107.71 (10.75)	108.61 (11.02)
ii) SBP Category				
Normal, n (%)	53 (81.54)	45 (84.91)	273 (85.85)	3142 (82.64)
Normal-high, n (%)	8 (12.31)	3 (5.66)	27 (8.49)	328 (8.63)
Stage 1 hypertension, n (%)	4 (6.15)	5 (9.43)	17 (5.35)	308 (8.10)
Stage 2 hypertension, n (%)	0 (0.00)	0 (0.00)	1 (0.31)	24 (0.63)
iii) Mean DBP (SD) / mmHg	64.65 (5.75)	67.19 (7.38)	64.86 (7.51)	65.31 (7.44)
iv) DBP Category				
Normal, n (%)	62 (95.38)	46 (86.79)	292 (91.82)	3494 (91.90)
Normal-high, n (%)	3 (4.62)	3 (5.66)	21 (6.60)	197 (5.18)
Stage 1 hypertension, n (%)	0 (0.00)	4 (7.55)	5 (1.57)	100 (2.63)
Stage 2 hypertension, n (%)	0 (0.00)	0 (0.00)	0 (0.00)	11 (0.29)

**Table 5:** Multivariable logistic regression for SBP and DBP, adjusting for age, bmi and cardiac disease (*n* = 4225)

	OR (95% CI)	Std Err	*P*-value
Normal SBP (base outcome)			
Raised SBP			
MPH < 12 months	1.09 (0.56–2.12)	0.37	0.796
MPH ≥12 months	0.91 (0.41–1.99)	0.36	0.807
ADHD Control	0.65 (0.46–0.92)	0.12	0.016^¥^
Non-ADHD Control	Reference		
Normal DBP (base outcome)			
Raised DBP			
MPH < 12 months	0.55 (0.17–1.77)	0.32	0.315
MPH ≥12 months	1.80 (0.80–4.06)	0.75	0.159
ADHD Control	0.88 (0.57–1.35)	0.19	0.554
Non-ADHD Control	Reference		

^¥^ significant

## Discussion

This study investigated naturalistic data from a cohort of children collected within a large nation-wide survey in Germany. The aim of this study was to explore growth and cardiovascular outcomes across different patient groups, in particular to evaluate the hypothesis that boys with ADHD and current MPH use show a higher percentage of having low BMI, having low height, and increased systolic and diastolic BP compared to controls.

### Height and BMI

Our analysis was unable to confirm our hypotheses that MPH use is also associated with low height (height ≤ 3rd percentile).

Several studies in children with ADHD have investigated associations between ADHD medication and height. Hanć and colleagues reported that the height of drug-naïve boys with ADHD was not significantly different from the norm [17]. Zhang and colleagues examined the impact of long-term treatment of MPH on height and weight of school age children with ADHD and reported a small but significant deceleration of height velocity, the magnitude of which was related to the duration of treatment [36].

Our analyses of the KiGGS dataset overall showed that MPH use in boys with ADHD was associated with low BMI. This effect was observed in the younger boys aged 6–10 years with MPH use of less than 12 months (OR: 4.52, 95% CI: 1.54–13.28, *p* = 0.006). There was a trend towards an effect in the boys aged 11–15 years with MPH use ≥12 months (OR: 3.59, 95% CI: 1.06–12.22, *p* = 0.04). Boys aged 6–10 years with MPH use ≥12 months and boys 11–15 years with MPH use less than 12 months did not significantly differ from the non-ADHD control group in that respect. Several considerations might help to interpret these findings:

A *fast* weight lost within a short amount of time (weeks or months) can raise concerns and lead to discontinuation of medication (in particular in younger children). This could explain why low BMI was more often in boys 6–10 years of age with MPH use of less than 12 months compared to controls, but not in the group with an MPH use ≥12 months (as this sample was reduced by the number of those who had to discontinue due to earlier weight loss).

However, we found the opposite effect in pubertal/post-pubertal boys. This might be due to a higher proportion of *slow* weight loss resulting in low BMI after more than one year of treatment. One reason for this might be a less severe post-pubertal effect of MPH on weight either due to metabolic reasons or due to a better way of coping with reduced appetite in this age group (eating larger meals in the later evening). These findings should be interpreted with caution due to the small numbers and wide confidence intervals and further research is required to verify these considerations.

The relationships between ADHD, methylphenidate use and growth appear to be complex. Some evidence suggests that there is a significant association between ADHD per se and obesity/overweight among patients, however this relationship is not consistent across studies [37]. Schwartz and colleagues used longitudinal health record data to examine associations between ADHD diagnosis and stimulant use on BMI trajectories throughout childhood and adolescence [15]. Their findings suggested that ADHD during childhood not treated with stimulants was associated with higher childhood BMIs whereas BMI was reduced in those whose ADHD was treated with stimulants but for this group there was a rebound later in adolescence to levels above those for children without stimulant use or without a history of ADHD [15]. Taking into account findings for both parameters (height and BMI) our data suggests that MPH may have more of an effect on weight than on height, a finding also highlighted by previous studies [10, 38].

### Cardiovascular outcomes

Our analyses of the KiGGS dataset were unable to confirm the hypotheses that MPH use in boys with ADHD is associated with increased systolic and/or diastolic BP (≥90th percentile).

A review of the cardiovascular effects associated with MPH published in 2014 reported that changes in BP across the eight included studies (*n* = 970) ranged from − 4.3 to + 21.2 mmHg for mean SBP, and − 4.7 to + 4.0 mmHg for mean DBP. When duration of treatment was examined, the authors concluded that short-term use (≤6 months) of MPH was associated with small increases in BP that were not statistically significant whereas the data for MPH ≥ 6 months’ duration provided “a more mixed picture ...with some decreases in SBP and DBP… reported. However, it should be noted that these longer-term results were not corrected for age” [39].

Clinicians need to be cognisant however of the effects that even small changes in BP can have. Meta-analytic data from adult studies at ages 40–69 years estimate that each difference of 20 mmHg in usual SBP or 10 mmHg in DBP is associated with a two-fold increase in the rate of death from ischaemic heart disease and other vascular causes and more than a two-fold increase in the death rate from stroke [40]. In children, the exact level and duration of elevated BP that causes target organ damage are not established. However, there is increasing evidence that even very small elevations in BP can have long-term adverse effects on vascular structure and function [41].

### Limitations

There are a number of limitations which should be considered in the current study. This study is cross-sectional in nature and thus inferences of causality cannot be made. For example, confounding factors (e.g. genes) may influence the likelihood of being treated via symptom severity and also influence body weight. Follow-up data were not available which would be particularly important for assessment of growth trajectories in children. However, analyses of growth outcomes were conducted stratified by age (< 11 years and ≥ 11 years) to account for the correlation between height velocity, growth spurts and pubertal maturation. The ADHD control group consists of children with an ADHD diagnosis but no current use of MPH. However, data were not available as to whether these children were stimulant-naïve or had prior exposure to MPH. The data were not gathered for the primary purpose of investigating the current research question and such secondary analyses cannot gather additional data on prior exposure to MPH; it is acknowledged that this potential contamination effect may attenuate differences among the groups. Information on dosing of MPH was not complete for all patients and, therefore, could not be included in the analyses. Identifying the ADHD cohort within the KiGGS sample was done by parent report of an ADHD diagnosis that had been made by a physician or psychologist. Due to the large number of children included and the wide range of competing physical, psychological and social health indicators which comprised the dataset, a full psychiatric diagnostic interview was not possible. However, other studies which have examined prevalence of ADHD in the KiGGS cohort have obtained a rate of 4.8% which is well in line with other population-based estimates [42]. The numbers of children within the two MPH groups were low and thus this may have contributed to a lack of precision around the estimates reported and the potential for Type II errors should be considered. Selection bias may be an issue within the MPH ≥ 12 months group insofar as individuals in whom adverse effects emerge may be less likely to persist with MPH treatment in the long-term. Finally, although the original study sample was assessed over ten years ago and the total number of children and adolescents in Germany decreased since then (as in many Western countries), we do not feel that this impacts on the findings of the current study.

## Conclusion and implications for practice

When looking at the balance between risks and benefits of MPH treatment for ADHD, it needs to be considered that ADHD itself is associated with a broad range of psychosocial impairments such as; school failure, parental and family conflict, social rejection by peers, low self-esteem, higher risk for delinquent behaviour, smoking and substance use disorders. Adverse outcomes continue into adolescence and adulthood to include academic and vocational underachievement, reduced occupational functioning, emotional dysregulation, anxiety, depression, unemployment and suicide attempts, higher rates of traffic accidents, unwanted pregnancies, preterm mortality [43].

Secondly, findings from national registry studies indicate that the use of medication, particularly stimulants, reduces the risk of accidents and trauma-related emergency department admissions and might have protective effects on substance abuse, suicidality and delinquent behaviour [44–46].

We have used cross-sectional data from a German national representative sample to identify adverse growth and cardiovascular outcomes associated with MPH. Effects on BMI were observed among the MPH cohorts; clinicians should discuss with all patients and their parents the potential effects on growth and balance these effects with the outcomes of not treating ADHD symptoms. Particular attention should be paid to those patients in the lower growth percentiles. Serious concerns about growth warrant referral to a paediatric endocrinologist or growth expert [24]. This study is also one of the first to present categorical data on hypertension in patients taking MPH. While these data did not highlight significant differences overall between MPH cohorts and controls with respect to raised BP, the data do not preclude clinically significant BP increases in single cases, and so it is recommended to check BP and pulse prior to initiating MPH treatment and to monitor regularly throughout treatment. Sustained elevated BP before or during MPH treatment requires assessment and treatment. Further details on the management of adverse effects of medication for ADHD are presented by Graham and colleagues [24], Cortese and colleagues [47] and the 2018 NICE Clinical Guideline [NG87] [4].

## Abbreviations

ADHD: Attention deficit hyperactivity disorder
AMP: Amphetamine
BMI: Body Mass Index
BP: Blood Pressure
DBP: Diastolic Blood Pressure
KiGGS: The German Health and Examination Survey for Children and Adolescents
MPH: Methylphenidate
OR: Odds Ratio
SBP: Systolic Blood Pressure
SD: Standard Deviation
